# Radiation-Induced Valvular Heart Disease: A Narrative Review of Epidemiology, Diagnosis and Management

**DOI:** 10.3390/jcdd13010001

**Published:** 2025-12-19

**Authors:** Andreea-Mădălina Varvara, Cătălina Andreea Parasca, Vlad Anton Iliescu, Ruxandra Oana Jurcuț

**Affiliations:** 1 Faculty of Medicine, Carol Davila University of Medicine and Pharmacy, 050474 Bucharest, Romania; catalina.parasca@umfcd.ro (C.A.P.); vlad.iliescu@umfcd.ro (V.A.I.); ruxandra.jurcut@umfcd.ro (R.O.J.); 2 Cardiology Department, Emergency Institute for Cardiovascular Diseases, 258 Fundeni Street, 022328 Bucharest, Romania

**Keywords:** radiation-induced VHD, mediastinal radiotherapy, cancer survivors

## Abstract

Mediastinal radiotherapy plays a central role in the treatment of several malignancies, particularly Hodgkin lymphoma and breast cancer. However, exposure to thoracic radiation is associated with long-term cardiovascular complications, among which valvular heart disease (VHD) is increasingly recognized. Radiation-induced VHD typically presents after a latency period of 10–20 years and is characterized by progressive valve fibrosis, thickening, and calcification, most commonly affecting the left-sided valves. Management of radiation-induced VHD generally follows standard guidelines but remains challenging due to extensive calcification and coexisting radiation-related cardiac or pulmonary injury. A history of thoracic radiotherapy is associated with increased perioperative risk and may negatively impact surgical outcomes, which often alters the risk–benefit balance and favors less invasive therapeutic approaches. Advances in the transcatheter approach have expanded treatment options for this high-risk population; however, data on long-term outcomes remain limited. Evolving dose-reduction techniques, such as deep-inspiration breath-hold, intensity-modulated radiotherapy, and proton therapy, together with predictive dosimetric models, aim to minimize future cardiac toxicity. Given the delayed onset and progressive nature of radiation-associated VHD, structured long-term surveillance is essential to enable early detection and timely intervention in cancer survivors at risk.

## 1. Introduction

Cardiovascular disease is a significant contributor to mortality among cancer patients, with over 10% of deaths attributed to cardiovascular causes rather than to the malignancy itself [1]. An increased risk of cardiovascular complications can occur both during oncologic therapy and after its completion, highlighting the need for proactive follow-up of cancer survivors (CSs).

Radiation therapy (RT) is associated with a high risk of cardiovascular complications, affecting any cardiac structure within the radiation field. Among survivors of Hodgkin lymphoma (HL), the most commonly observed conditions are coronary artery disease and valvular heart disease (VHD), occurring in approximately 17–23% and 8–17% of patients, respectively [2].

As a late complication in CS, VHD usually occurs after more than 10 years following RT, with the most frequently affected being the left-sided heart valves [3]. Due to the prolonged latency period, it is crucial for clinicians to be knowledgeable about the risk factors associated with radiation-induced heart disease and the most frequent associations [4], as well as the necessity for regular proactive follow-up and, when indicated, timely consideration of treatment options for VHD.

This narrative review is based on research identified through narrative searches in PubMed, and MEDLINE, using terms related to mediastinal radiotherapy, radiation-induced valvular heart disease, and cancer survivors.

## 2. Epidemiology

Mediastinal radiotherapy (MRT) is employed as part of the therapeutic protocol for various malignancies, including Hodgkin’s lymphoma, non-Hodgkin’s lymphoma, breast cancer, lung cancer, and esophageal cancer [3,5].

After MRT, VHD can more frequently involve the left-sided than the right-sided valves, regardless of the radiation dose, with valvular regurgitation being more prevalent than stenosis [6]. Irrespective of severity grading, aortic regurgitation exhibits the highest prevalence (38.2%), followed by mitral regurgitation (36.7%) and tricuspid regurgitation (20.4%), while pulmonary regurgitation has the lowest prevalence (9%) (Table 1). Regarding aortic stenosis (AS), its prevalence was determined to be 16.3%, compared to 4% for mitral stenosis [7].

Exposure to thoracic RT at a younger age is associated with a substantially higher long-term incidence of radiation-induced VHD. Data from the Childhood Cancer Survivor (CCS) study, which included patients irradiated before age 21, showed that those treated during cardiac development are particularly susceptible to late valvular degeneration, consistent with the concept of age-related cardiac radiosensitivity. After nearly three decades of follow-up, the 35-year cumulative incidence of clinically significant VHD reached 1.2%, whereas no severe valvular events occurred in non-irradiated controls [8]. Furthermore, evidence from a large pediatric cohort of over 40,000 childhood cancer survivors, with a median age at diagnosis of 6 years and a median follow-up of 25 years, showed that those irradiated before the age of 10 exhibited nearly twice the cumulative incidence of heart failure compared with those treated during adolescence, reflecting the global susceptibility of irradiated cardiac tissues. Importantly, this cohort evaluated symptomatic heart failure rather than valvular heart disease, and, therefore, it cited here to illustrate the broader cardiac vulnerability following thoracic irradiation, not as evidence of VHD incidence [9].

**Table 1:** Reported frequencies of VHD in survivors of Hodgkin’s Lymphoma treated with mediastinal radiotherapy—overview of retrospective studies. * Studies reporting prevalence of VHD; ** Studies reporting incidence of VHD; † Mean dose; ‡ at least moderate severity; § percentage of affected valves; values refer to combined aortic and mitral valve regurgitation; ‖ Tricuspid and pulmonary regurgitation data were available for only 39 of the 47 patients. Mod, moderate; N/A, Not Available; RT, radiotherapy; VHD, valvular heart disease.

Study	RT–VHD Interval (Years)	Median Dose (Gy)	Patients with VHD (%)	Aortic Regurgitation		Aortic Stenosis		Mitral Regurgitation		Mitral Stenosis		Tricuspid Regurgitation		Pulmonary Regurgitation	
				Mild (%)	≥Mod (%)	Mild (%)	≥Mod (%)	Mild (%)	≥Mod (%)	Mild (%)	≥Mod (%)	Mild (%)	≥Mod (%)	Mild (%)	≥Mod (%)
Jesse M. Bijl et al. [7] *	16.5	40	61.3	26	12	4	12	25	12	2	2	15	36	9	0
Cutter et al. [10] **	23.3	37 †	4.8	3.4 ‡				2.2 ‡				0.49 ‡		N/A	
Galper SL et al. [11] **	16.1	40	6.1	N/A	15.2 §	N/A	13.3 §	N/A	47.6 §	N/A	0.9 §	N/A	22.8 §	N/A	N/A
Schellong et al. [12] **	19.5	25	3.96	2.88				1.68				0.72		0.36	
Wethal et al. [13] **	22	40	N/A	29.4	68.6	25.5	13.7	29.4	68.6	N/A	N/A	N/A	3.9	N/A	3.9
Adams MJ et al. [14] *	15.5	40	42.6	19		6		20.8		2		25.6 ‖		2.6 ‖	
Hull MC et al. [15] **	22	37	6.2	N/A	3.4 §	N/A	48.2 §	N/A	27.6 §	N/A	10.3§	N/A	10.3 §	N/A	N/A
Heidenreich P. et al. [16] *	15	43 †	29	21.1	5.1	4		35.7	3.4	N/A	N/A	14.6	1.4	6.8	0
Lund et al. [17] *	9	40.6	31	9.5	17.2	N/A	N/A	12.9	10.3	N/A	N/A	26.7	5.2	6	1.7

Radiation-induced VHD has an increased frequency with longer time intervals following RT. Galper et al. reported progressive development of valvular dysfunction in 1279 patients treated with RT for HL, with prevalence rates of 1.4% at 10 years, 3.3% at 15 years, and 5% at 20 years [11]. Likewise, Hull et al. observed rates of 1%, 4%, and 6% at the same intervals in a cohort of 415 HL patients [15]. Also, interval since RT plays an important role in the severity of VHD, with the highest prevalence observed beyond 20 years post-exposure. Heidenreich et al. highlighted this trend in a study which analyzed 294 asymptomatic survivors of HL. It was noted that the prevalence of mild and moderate to severe aortic regurgitation (3.4% and 1.1%) and mitral regurgitation (24% and 2.3%) was substantially lower in patients who had received RT within the past 10 years compared to those evaluated more than 20 years post-radiation exposure (aortic regurgitation: 45% and 15%; mitral regurgitation: 48% and 4.1%) [16].

Moreover, the likelihood of patients developing VHD increases progressively with higher radiation doses delivered to the heart. In a retrospective study of 1852 HL patients who underwent RT with two-dimensional techniques (between 1965 and 1995), researchers estimated the cumulative radiation dose to the affected valves and observed that for doses of ≤30, 31–35, 36–40, and >40 Gy, the frequency of VHD occurrence was 1.4, 3.1, 5.4, and 11.8 times higher, respectively [10]. Although these data are based on prescribed mediastinal doses, later cohorts using modern dosimetry have shown that radiation-related cardiac injury is also influenced by the proportion of cardiac volume exposed, suggesting that both the delivered dose and the irradiated volume contribute to the long-term cardiac tissue burden [9]. Evidence from these databases indicates that, even with mean heart doses (MHDs) of 5–15 Gy, the risk increased notably when at least half of the cardiac volume was irradiated [9].

Additionally, historical 2D MRT exposed the heart and valvular structures to substantially higher doses than contemporary techniques, as shown in Table 1, where a single asterisk denotes prevalence, and a double asterisk denotes incidence of VHD. These cohorts include classical HL survivors treated between 1961 and 2005, predominantly with mantle-field regimens, which frequently delivered mean or median heart doses of 25–40 Gy. Under these conditions, characterized by both high doses and large treatment fields, the observed exposures likely explain the highest VHD rates reported in these older RT eras. Variation in VHD rates across studies in Table 1 likely reflects differences in imaging modality and cohort selection. Thus, systematic echocardiographic screening of asymptomatic survivors detects a higher prevalence of subclinical valvular lesions [7], while studies based on clinical presentation tend to underreport mild cases due to selection bias [10,11].

In comparison, modern treatment approaches, such as three-dimensional conformal radiotherapy (3D-CRT), intensity-modulated radiation therapy (IMRT) or volumetric modulated arc therapy (VMAT), and image-guided methods like deep-inspiration breath-hold (DIBH), considerably reduced MHD and valvular doses by approximately 50–80% compared with 2D RT. These substantial dosimetric improvements indicate that absolute VHD risks for patients treated today are expected to be markedly lower than those observed in historical cohorts [18,19]. In a study of 42 patients with stage IIA HL treated with doxorubicin, bleomycin, vinblastine, and dacarbazine chemotherapy followed by 30 Gy RT using 3D-CRT and VMAT, the absolute risk of VHD was 2.5 and 1.14 times higher, respectively [20]. When using DIBH, lung expansion during inspiration moves the heart inferiorly and posteriorly, increasing the heart-to-chest distance and sparing the heart (and valves) from the high-dose field in both breast and mediastinal treatments. In a 2021 study, patients with left-sided breast cancer saw a significant reduction in MHD, decreasing from about 4.0 Gy in free breathing to about 2.4 Gy with DIBH [18]. This reduction was even more evident when the cancer was located in the upper mediastinum, where MHD dropped by about 50% with DIBH as the heart retracted from the radiation field [18].

As radiation techniques become more precise in shaping the dose to the tumor’s exact contours while sparing surrounding tissues, the link between MHD and the dose received by individual cardiac substructures becomes less predictable, as the distribution is strongly influenced by the geometry of the beam arrangement [19,21]. Proton therapy offers even lower exposure, typically under 2 Gy due to the Bragg peak effect [22], though its use is limited by cost and availability, and long-term outcome data are still needed. A study comparing proton and photon planning in mediastinal lymphoma showed that cardiac irradiation is distributed non-uniformly, reflecting the predominantly anterior or anterior-oblique beam arrangements. In the proton plans generated with a Monte Carlo algorithm, the pulmonic and aortic valves, which lie most anteriorly in the mediastinum, received the highest doses (mean ≈ 27–30 Gy and 9–13 Gy, respectively), whereas the mitral and tricuspid valves, located more posteriorly or laterally, showed lower and more variable values (mean ≈ 0.9–6.1 Gy and 1.8–10.2 Gy) [23].

## 3. Pathophysiology and Histopathology

Autopsy studies have shown that patients exposed to MRT exhibit fibrosis or thickening of cardiac valve leaflets with diffuse or focal distribution, as well as areas of calcification, although no inflammatory changes or neovascularization have been identified [24]. While the exact mechanisms underlying cardiac valve damage secondary to RT remain incompletely understood, we know that radiation can pathologically activate valvular interstitial cells and differentiate them into myofibroblasts and osteoblast-like cells. Consequently, radiation triggers fibrogenic growth factors, such as transforming growth factor β1, activates myofibroblasts, and stimulates collagen synthesis [25,26]. Also, it induces endothelial injury and microvascular dysfunction in cardiac tissue, initiating an inflammatory cascade and chronic ischemia. This avascular nature of irradiated valve components contributes to progressive valve fibrosis [25,27].

**Figure 1:**
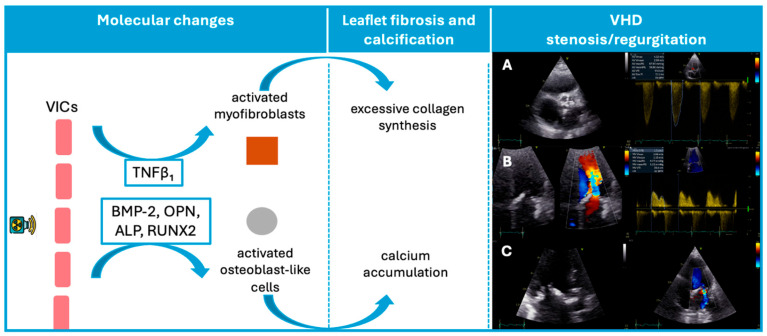
Schematic representation of the pathophysiological sequence from radiation exposure to valvular dysfunction. In this VHD panel, three illustrative cases are shown: (**A**) Severe aortic stenosis with marked annular and cusp calcification, resulting in a peak transvalvular velocity of 4.12 m/s. (**B**): Mitral valve thickening with pronounced leaflet fibrosis and calcification, associated with a mean diastolic gradient of 5.22 mmHg, consistent with moderate mitral stenosis. (**C**): Markedly thickened mitral leaflets with calcific involvement, and color Doppler demonstrating a moderate degree of mitral regurgitation. ALP, Alkaline phosphatase; BMP-2, Bone morphogenetic protein-2; OPN, Osteopontin; RUNX2, Transcription factor Runx2; VICs, valvular interstitial cells; VHD, valvular heart disease.

Consistent with this mechanism, Nadlonek et al. demonstrated that irradiation of aortic interstitial cells induced an osteogenic phenotype, with increased expression of osteogenic factors, including bone morphogenetic protein 2, osteopontin, alkaline phosphatase, and the transcription factor Runx2 (Figure 1) [28]. These factors may explain why patients exposed to RT frequently exhibit valve calcifications [28]. Similar to conventional degenerative valve disease risk factors, concomitant systemic factors, such as high lipid levels or chronic kidney disease, may accelerate valvular calcification after radiation [29,30].

As such, radiation-induced VHD starts as a degenerative process leading initially to valve retraction, which manifests as regurgitation, and progresses to thickening and calcification of valves, ultimately culminating in stenosis.

## 4. Risk Factors

**Figure 2:**
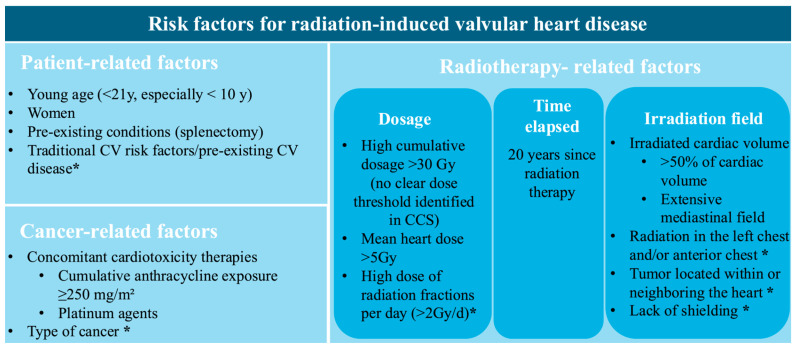
Risk factors for radiation-induced valvular heart disease. CV, cardiovascular; Gy, Gray; * Identified as a general risk factor for radiation-related heart disease, not specific to valvular pathology.

Physicians have to be aware of associated risk factors and the necessity of regular surveillance through serial, clinical, biological, and multimodality imaging due to the extended latency of radiation-induced VHD. Figure 2 provides a schematic representation of these risk factors. They are structured into categories associated with RT, cancer, and patient characteristics. There is a distinction between the ones connected to a higher risk of radiation-induced VHD and those acknowledged as common risk factors for radiation-induced heart disease.

Risk factors include factors related to radiation therapy, such as radiation dose [10], latency period [11,15], and irradiation field, which are discussed in detail in the Epidemiology Section. Notably, in the CCS study, higher mean radiation doses to cardiac valves were independently associated with the development of late VHD. No clear radiation dose threshold, below which valvular injury can be excluded, could be identified. Even mean doses as low as 5–9.9 Gy to valvular or ventricular substructures were associated with a more than fivefold increase in the risk of late VHD [8]. The extent of irradiated cardiac volume emerged as a strong determinant of radiation-induced VHD. In the PanCare/ProCardio cohort, survivors with >50% of the cardiac volume exposed to ≥5 Gy or >15 Gy had 15.9-fold and 18.1-fold higher odds of developing heart failure, respectively, highlighting the importance of cardiac dose–volume parameters in predicting cardiac tissue injury [31].

Cancer-related factors include the type and localization of the cancer, with HL, non-HL, breast cancer, and thoracic malignancies (such as lung and esophageal cancer) carrying a higher risk of developing radiation-induced heart disease [5]. The cumulative dose of anthracyclines is another important determinant in the development of VHD. In a study by van Nimwegen et al., it was observed that doses below 200 mg/m^2^ were not associated with a significant increase in risk, whereas doses of 200–325 mg/m^2^ and 350–880 mg/m^2^ were linked to a 1.5-fold and 3.3-fold higher risk, respectively [32]. In a large European cohort of CCSs, a cumulative anthracycline dose of ≥ 400 mg/m^2^ was independently associated with VHD, after adjustment for mean heart radiation dose, showing a 3.8-fold higher risk. Although a risk estimate for co-exposure was not reported, patients receiving both MRT > 15 Gy and ≥250–400 mg/m^2^ cumulative anthracycline doses appeared to have the greatest overall burden of VHD [31]. Furthermore, platinum-based chemotherapy was not independently associated with VHD after adjustment for RT and anthracycline exposure, but may potentiate radiation-induced valvular injury through endothelial and microvascular damage [31].

Patient-related risk factors, including younger age during RT (<21 years), cardiovascular risk factors, and previous cardiovascular disease, are widely recognized as significant contributors to the development of radiation-induced VHD [6]. Splenectomy is associated with a significantly increased risk of VHD, particularly in CCSs and in individuals with certain hematological conditions. Given the spleen’s role in regulating immune status and maintaining normal coagulation balance, its absence leads to higher proinflammatory and prothrombotic factors [33]. These mechanisms may accelerate valvular fibrosis and calcification, processes in which chronic inflammation and immune imbalance play an important pathogenic role [34]. Accordingly, in a cohort of 5286 CCSs, splenectomy conferred a hazard ratio of 8.6 for symptomatic VHD, with a cumulative incidence at age 40 increasing to 2.7%, compared with 0.4% in individuals without splenectomy [35]. Also, research on sex-related factors in VHD has indicated that women may experience more severe manifestations, as they exhibit increased aortic valvular fibrosis and greater calcification of the mitral annulus [36]. Among patients who underwent RT for HL, the incidence of cardiovascular events and mortality was nearly four times higher in women than in men [5,37].

## 5. Diagnostic Tests

Regardless of the underlying etiology, the diagnosis and severity of VHD in cancer patients are assessed using the same criteria as in patients without cancer, according to the guidelines published by the European Society of Cardiology (ESC) in 2025 [38].

Transthoracic echocardiography (TTE) is the first-line imaging modality for diagnosing VHD. According to current cardio-oncology guidelines, TTE is recommended for all patients at high or very high risk of developing cardiotoxicity, serving as a baseline assessment before initiating cancer treatment [39]. During treatment, the frequency of TTE monitoring is adjusted based on the patient’s cardiovascular toxicity risk, as determined by the HFA-ICOS risk score and the specific oncologic therapy administered [39]. Accordingly, in high- or very-high-risk patients, TTE assessments are generally recommended every 3 months, or even every two chemotherapy cycles during anthracycline therapy, while less frequent follow-up is advised for lower-risk patients [39]. Among patients who have undergone RT, VHD is frequently identified incidentally or after the onset of heart failure symptoms, usually manifesting as a late-stage complication.

**Figure 3:**
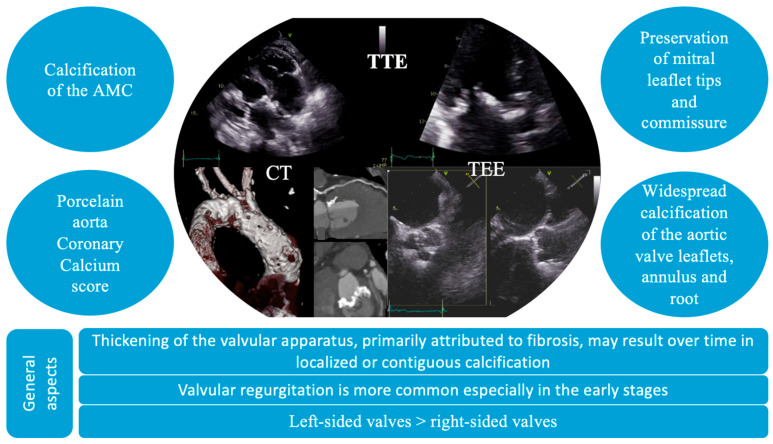
Particular imaging characteristics of radiation-induced valvular heart disease on multimodal imaging. AMC, aorto-mitral curtain; CT, computer tomography; TEE, transesophageal echocardiography; TTE, transthoracic echocardiography.

The diagnosis of VHD includes the identification of structural abnormalities of the valves, a comprehensive assessment of valvular function, and an evaluation of the hemodynamic and functional impact on ventricular performance. In these patients, distinctive echocardiographic features have been identified, characterized by diffuse valvular thickening, primarily attributed to fibrosis and either localized or contiguous calcification [40]. These structural alterations result in restricted leaflet mobility, initially presenting as valvular regurgitation, which may progressively advance to stenosis [4]. Valvular thickening and calcification induced by radiation tend to be widespread, affecting adjacent structures, such as the valve annulus, aortic root, subvalvular apparatus, and aorto-mitral curtain. In particular, mitral valve disease typically spares the leaflet tips and commissures. Moreover, it can often affect multiple valves (Figure 3) [4]. Also, the thickness of the aorto-mitral curtain (AMC) warrants special attention due to its prognostic significance, with evidence showing that an AMC measurement greater than 6 mm independently correlates with increased mortality in patients with radiation-induced cardiac disease [41].

Furthermore, the role of TTE should not be overlooked in detecting other cardiac complications secondary to RT. The use of deformation imaging (strain) has proven essential for identifying subclinical myocardial dysfunction, as demonstrated by the BACCARAT study, which reported that nearly half of the patients developed varying degrees of cancer therapy-related cardiac dysfunction two years after RT [42]. TTE is also valuable in assessing pericardial involvement, which most commonly presents as pericardial thickening, but may also manifest as constrictive pericarditis [43,44].

Transesophageal echocardiography (TEE) is a valuable alternative to TTE, which can have poor acoustic windows or interference from calcifications, allowing a more precise assessment of both anatomical structures and valve function. Additionally, TEE is essential in preprocedural planning, assisting in determining the suitability of patients for transcatheter or surgical interventions [6]. However, in these patients, TEE carries an increased risk due to radiation-induced structural changes in the esophageal wall, such as fibrosis and rigidity [45].

Also, if echocardiography is unavailable or inconclusive, cardiac magnetic resonance imaging (CMR) should be considered with a class IIa indication [39]. CMR is widely recognized as the most reliable and comprehensive modality for assessing cardiac chamber size, volumes, function, morphology and valvular anatomy. Additionally, CMR can provide complementary information, such as the evaluation of myocardial fibrosis [46].

Other imaging modalities can also provide valuable insights into the secondary effects of RT, including computed tomography (CT) and functional imaging techniques for detecting myocardial ischemia, such as stress echocardiography, perfusion CMR, or nuclear myocardial perfusion imaging [47]. Cardiac CT is a high-resolution imaging modality which can provide three-dimensional visualization of valve morphology and associated cardiac and extracardiac structures. As such, it can quantify calcific burden through calcium scoring of individual valve components, making it an invaluable tool for risk stratification and procedural planning [48,49,50]. In addition, CT imaging or CT angiography, which are frequently performed in oncology patients for cancer staging, can be useful in assessing coronary artery disease through coronary artery calcium score, porcelain aorta, pericardial thickening, or calcification, and even the detection of cardiac or mediastinal masses [47]. Consequently, patients with specific conditions such as porcelain aorta or severe annular calcification have a high surgical risk and tend to favor a transcatheter strategy. Conversely, CT may identify anatomical features such as unfavorable annular dimensions, coronary ostial proximity, or inadequate peripheral access that can rule out a transcatheter intervention [51].

## 6. Management and Follow-Up

Given the late onset of radiation-induced VHD and the improving long-term survival of cancer patients, extended surveillance strategies are needed and timely assessment for VHD has become increasingly important. The main recommendations are based on the ESC guidelines and the International Cardio-Oncology Society (ICOS) expert consensus statement, both of which propose protocols for the screening and diagnosis of radiation-induced VHD. TTE continues to be the preferred screening modality.

Pretreatment cardiovascular evaluation is essential in RT planning, as it helps stratify cardiotoxicity risk and guide the implementation of cardio-protective measures. For baseline risk assessment, both the ESC guidelines and the ICOS consensus recommend TTE (or CMR if TTE is inconclusive), especially in high-risk patients with pre-existing CV disease [39,52]. Furthermore, thoracic CT scans performed for cancer staging may enhance cardiovascular risk assessment by enabling coronary calcium scoring as an indicator of coronary artery disease in selected patients [52].

**Figure 4:**
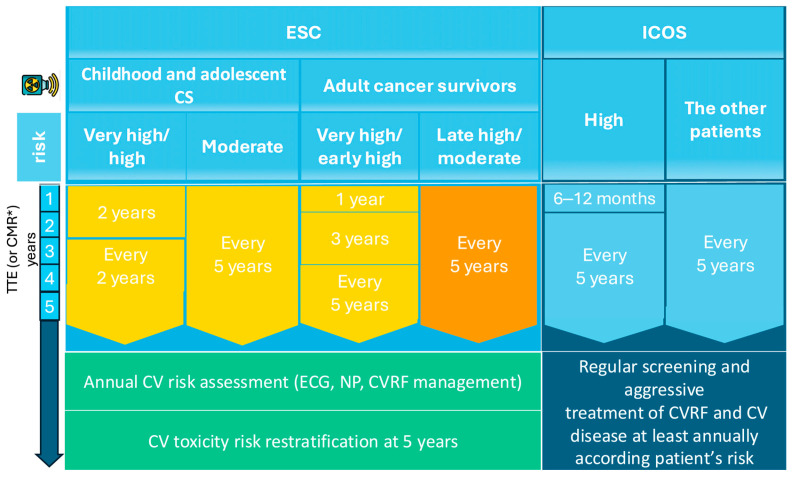
Follow-up of VHD secondary to RT. * if TTE is inconclusive; CMR, Cardiac magnetic resonance; CV, cardiovascular; CVD, cardiovascular disease; CVRFs, cardiovascular risk factors; ESC, European Society of Cardiology; Gy, Gray; ICOS, International Cardio-Oncology Society; MHD, mean heart dose; NPs, natriuretic peptides; TTE, Transthoracic echocardiography.

Expert consensus guidelines emphasize periodic echocardiographic surveillance for all CSs who underwent RT and have at least moderate cardiovascular risk, with imaging frequency tailored to individual risk stratification (Table 2). According to the recent ESC guidelines for VHD, periodic TTE surveillance is recommended for patients at risk, starting approximately 10 years after MRT, with follow-up examinations every 5 years thereafter [38]. The ESC Cardio-Oncology guidelines further provide detailed recommendations that stratify CSs into the following two categories: those treated for cancer during childhood or adolescence, and those treated during adulthood. The ESC recommends that survivors of childhood or adolescent cancer undergo cardiac assessment every two years if they are high risk, or every five years if they are moderate risk. For adult CSs, the ESC advises that very-high- and early high-risk patients undergo TTE one year after completing treatment, followed by imaging at three and five years, and then continue surveillance at five-year intervals; moderate- and late high-risk CSs are generally evaluated every five years (Figure 4) [39].

In comparison, the ICOS advocates for a stricter cardiac screening, recommending TTE within 6–12 months for high-risk CSs and at least one echocardiographic evaluation within five years for any patient whose cardiac structures were exposed to radiation [52]. Moreover, several studies suggest starting echocardiography surveillance 10 years post-radiotherapy, or earlier if significant signs or symptoms are present, with subsequent evaluations every five years in patients without valvulopathy [53,54]. If new VHD is detected, management should follow existing guidelines.

For effective long-term monitoring, CSs treated with RT should undergo annual cardiovascular assessments, including electrocardiogram, natriuretic peptide measurement, and active management of CV risk factors. Also, reassessment of cardiotoxicity risk is recommended five years post-treatment to guide individualized long-term follow-up strategies [39].

## 7. Treatment

Therapeutic decisions regarding CSs with a diagnosis of significant radiation-induced VHD should be made within a multidisciplinary heart team. Since they require an evidence-based and often individualized approach, the heart team needs to include experts in both cardio-oncology and structural heart disease.

Although there are no specific recommendations regarding the optimal timing of intervention in this subgroup, therapeutic decisions should follow current clinical guidelines for the management of VHD [55,56]. Most patients with radiation-induced VHD are long-term survivors with sustained cancer remission; therefore, therapeutic decisions should primarily take into account the patient’s age, comorbidities, procedural risk, and individual preferences, rather than oncologic prognosis or life expectancy [38,39].

Beyond VHD, patients with a history of MRT frequently develop associated cardiovascular, mediastinal, and pulmonary sequelae, such as ostial coronary artery stenosis, pericardial involvement, or mediastinal and pulmonary fibrosis, which may have major implications for both diagnostic assessment and therapeutic strategy. Therefore, pretreatment assessment should not be limited to valvular evaluation alone.

In practice, VHD management often becomes more complex when additional clinical scenarios interfere with timely diagnosis and intervention. Consequently, the outcomes of cardiac surgery are adversely affected by radiation-related pulmonary fibrosis. In a cohort of 230 patients who underwent cardiac surgery after MRT, those with extensive fields had higher rates of respiratory complications, 24% and 9.6%, respectively, and higher in-hospital mortality, 13% and 2.4%, respectively, compared with patients with only tangential exposure [57].

Another complex clinical scenario is the presence of severe coronary artery disease, which is more frequent in patients with prior thoracic radiotherapy and often involves ostial or proximal lesions [58]. In a study of 172 patients with severe radiation-associated aortic stenosis (AS) undergoing surgical valve replacement, 27% required concomitant coronary artery bypass grafting (CABG) and 34% required additional aortic procedures, reflecting the extent of mediastinal fibrosis and calcification [59]. Such combined pathology complicates procedural planning and may necessitate revascularization before or alongside valve intervention, with direct implications for the timing and choice of therapeutic strategy.

Reoperative cardiac surgery in patients with radiation-induced VHD represents another circumstance that can markedly influence both the management strategy and the timing of intervention. In a cohort of 261 patients, resternotomy was required in 18% and was associated with markedly higher operative mortality than primary surgery, with rates of 17% and 3.7%, respectively, and worse long-term survival, with an adjusted hazard ratio for death of 3.19 [60]. These risks explain why patients with a previous cardiac operation on an irradiated chest are often discussed earlier in a multidisciplinary team, and why less invasive or transcatheter approaches are frequently considered when feasible.

A clinical scenario that may affect the management and timing of VHD is reduced access to specialized care during phases of constrained healthcare capacity, such as the COVID-19 pandemic. In a cohort of 315 patients who underwent TAVI, the interval between diagnosis and intervention increased to 56.9 ± 68.3 days compared with 37.7 ± 25.4 days before the pandemic, and a higher proportion of patients presented in NYHA class III–IV [61]. Despite these differences, 30-day mortality and major complications remained unchanged, indicating that reorganized clinical pathways can preserve procedural safety even when access to healthcare is restricted [61].

According to the patient’s risk profile and clinical presentation, a comprehensive pre-treatment assessment is warranted, typically including coronary angiography, computed tomography angiography [62], and pulmonary function testing [63]. Additionally, it should be noted that commonly used scores for calculating surgical risk, such as the initial version of the Society of Thoracic Surgeons Predicted Risk of Mortality (STS-PROM) and the European System for Cardiac Operative Risk Evaluation (EuroSCORE II), may underestimate the risk associated with surgery in CSs due to specific complications induced by RT, such as pericardial calcification, aortic calcification, increased risk of bleeding, impaired tissue healing, and secondary pulmonary fibrosis [39]. As a result of its substantial impact on surgical mortality, a history of MRT has been incorporated into the STS risk assessment model for patients undergoing cardiac surgery [64].

### 7.1. Aortic Valve

The aortic valve is the most frequently affected structure in radiation-associated VHD, with aortic regurgitation being the most common early manifestation; however, patients often present with severe calcific aortic stenosis at the time intervention is required. In adult CSs, radiation-induced AS tends to present earlier than idiopathic degenerative AS; cases in the fifth or sixth decade are not uncommon, especially in those irradiated in childhood or adolescence [65]. The ESC Guidelines for the management of VHD recommend transcatheter aortic valve implantation (TAVI) in older patients (≥70 years), as well as in those with high surgical risk or in patients deemed unsuitable for surgery, regardless of a prior history of MRT [38]. The ESC cardio-oncology guidelines provide more permissive recommendations for patients with symptomatic severe aortic stenosis and a history of MRT, supporting the use of TAVI in individuals at intermediate surgical risk [39]. Furthermore, guidance favoring TAVI in this population was already incorporated into the 2021 ESC Guidelines for the management of VHD, which include porcelain aorta and prior chest radiation among the anatomical and clinical factors supporting the use of TAVI [55].

In patients with a history of MRT and a high or prohibitive surgical risk, especially when important coronary artery disease is present, the American guidelines recommend a combined approach, using TAVI with percutaneous coronary intervention, due to its lower procedural morbidity and improved short-term outcomes compared to surgical alternatives [66].

Surgical aortic valve replacement (SAVR) carries an increased operative risk, with mortality rates reported to be up to five times greater than expected, due to factors such as mediastinal fibrosis and extensive aortic calcification, which make surgical access and the use of cardiopulmonary bypass difficult [67]. Furthermore, contemporary comparative data offer additional insight. In the cohort reported by Zhang et al., all patients had a history of MRT, and unadjusted 30-day mortality was 1.8% after TAVI compared with 9.1% after SAVR. Disabling stroke occurred in none of the TAVI patients and in 3.6% of those undergoing SAVR, and pacemaker implantation rates were similar, at 14.5% and 7.3%, respectively. The only clinical outcome parameter that differed significantly between the groups was the incidence of atrial fibrillation, which occurred in 32.7% of SAVR patients compared with 3.6% of those treated with TAVI [67].

If TAVI is not feasible due to technical or anatomical limitations, or if the patient has low to intermediate surgical risk, SAVR remains a valid option, even for patients with a history of MRT. Also, when concomitant procedures such as coronary artery bypass grafting (CABG) are needed, SAVR is preferred when indicated and technically feasible [66]. In younger patients undergoing SAVR, mechanical valves are often selected because of their durability, which lowers the likelihood of requiring a second operation. This consideration is particularly relevant in individuals with prior MRT, who, in this context, have a higher operative risk. Evidence shows that operative mortality among patients with a history of MRT is significantly higher, reaching approximately 17% in those undergoing reoperation and 3.7% in those undergoing their first cardiac surgery, in contrast to a 2.3% mortality rate observed in nonirradiated individuals [60].

In more complex cases where both the aortic and mitral valves require replacement, the Commando procedure, consisting of double valve replacement with reconstruction of the aorto-mitral curtain, should be considered. Additionally, hybrid strategies, including off-pump CABG followed by TAVI, may offer a tailored solution in selected patients. Optimal treatment decisions should be guided by a multidisciplinary heart team, carefully balancing anatomical considerations, comorbidities, and patient preferences [66].

### 7.2. Mitral Valve

Mitral regurgitation is the most frequent manifestation of radiation-induced mitral valve disease, and it can appear within the first decades after exposure. With time, a combined stenotic and regurgitant pattern may develop due to progressive calcification of the mitral annulus and leaflet base, leading to reduced leaflet mobility [40]. The mitral valve has a higher radiosensitivity than the aortic valve, with radiation doses of ≥30 Gy being associated with a significant increased risk of late valvular damage [31].

Prior MRT is strongly associated with accelerated mitral annular calcification (MAC), with more frequent involvement of the anterior annulus and the AMC, whereas the degenerative etiology usually affects the posterior annulus [68]. Also, proximity to the aortic valve and ostia of the coronary arteries, as well as features such as circumferential annular calcification, extension into the LV outflow tract, and involvement of the aorta, may complicate both surgical and transcatheter interventions, therefore requiring careful pre-procedural assessment and planning [69]. Moreover, the AMC is not only an imaging marker of radiation-associated fibrosis, but also a major determinant of surgical risk. In a cohort of surgically treated patients with radiation-associated cardiac disease, an AMC thickness of at least 6 mm independently predicted long-term mortality, with a hazard ratio of 5.75 even after adjustment for preoperative risk scores. These findings indicate that AMC thickening reflects a more advanced and diffuse fibrotic substrate, and its presence should be considered in treatment planning [41].

Intervention should follow standard guidelines, with treatment decisions focused on selecting between surgical mitral valve repair or replacement and transcatheter approaches. The ongoing radiation-induced changes make surgical valve repair a challenging option. In a surgical series of 22 patients, 32% experienced recurrent dysfunction, and half of early survivors required reoperation [70]. For this reason, surgical valve replacement is often preferred in these patients, although in a study of 146 patients, 77% underwent replacement and 23% underwent repair, with no significant difference in survival between the two groups [71]. Moreover, surgical treatment of radiotherapy-related MR carries high operative risk; in the same cohort, five-year survival was only 55%, compared to 95% for degenerative mitral disease surgery [71].

Recent data show that transcatheter interventions, including transcatheter edge-to-edge repair (TEER) and transcatheter mitral valve replacement (TMVR), are more frequently performed in patients with prior MRT, with similar in-hospital outcomes to non-radiated patients [72]. In a study of more than 810,000 transcatheter structural interventions, patients with previous MRT were more likely to undergo TEER (70 vs. 35 procedures per 100,000 hospitalizations) and TMVR (11 vs. 4 per 100,000), and adjusted in-hospital mortality and major procedural complications did not differ significantly between radiation and non-radiation groups [72]. These findings indicate that transcatheter mitral therapies are feasible and safe in appropriately selected patients with irradiated anatomy.

In clinical practice, TEER may offer short-term benefit in high-risk patients, though the higher risk of mitral stenosis in this patient category and disease progression limits its long-term durability [44]. Regarding leaflet grasping and transmitral hemodynamics after TEER, the post-procedural mean gradient was 4.5 ± 1.8 mmHg, with gradients above 5 mmHg in 3 of 15 patients [73]. During follow-up, three patients developed moderate mitral stenosis and one progressed to a valve area of 1.4 cm^2^ at 48 months, showing that transmitral obstruction can occur in about 20% of carefully selected RT-related MR patients and may progress late despite an initially acceptable gradient [73]. TMVR is an evolving therapy. Some case reports [74] demonstrate feasibility in these patients, and it is typically considered only if the patient is not a candidate for surgery and other options (like TEER) are unsuitable. The thickened MAC and stiff valvular tissue, often with extensive AMC involvement, can predispose to narrowing of the left ventricular outflow tract (LVOT) once a device is implanted [75]. LVOT obstruction is a significant procedural concern in TMVR, occurring in approximately 9.7% of patients in the MITRAL trial despite careful pre-procedural CT screening [76].

## 8. Research Gaps and Future Directions

Several important research gaps remain in the understanding and management of radiation-induced valvular disease. CT-based or AI-assisted dose prediction for individual cardiac substructures needs to be better integrated into risk assessment at the time of radiotherapy planning. Advanced imaging biomarkers, including quantitative valve calcium burden and fibrosis mapping, may help anticipate rapid progression and guide earlier referral to structural intervention teams.

Long-term outcome data for transcatheter valve therapies in irradiated patients are needed to clarify durability, progression of multivalvular disease, and the interplay between MAC distribution, LVOT obstruction risk, and procedural feasibility. Addressing these uncertainties will require prospective registries dedicated to structural interventions in radiation-induced VHD, including systematic data collection on TAVI, TEER, and TMVR in this high-risk population. Also, standardized imaging definitions for mitral and aortic calcification, particularly regarding aorto-mitral curtain thickening and subannular involvement, are lacking and hinder comparison across studies.

## 9. Conclusions

Radiation-induced VHD represents a growing concern among cancer survivors, often presenting decades after treatment. Given the high surgical risk, individualized and multidisciplinary management is essential. While transcatheter approaches are less invasive and have shown favorable early outcomes, further evidence is needed to establish their long-term safety and efficacy in this population. Ongoing clinical surveillance and advancements in RT planning play a critical role in enabling early diagnosis and guiding the optimal treatment.

## Figures and Tables

**Table 2:** Cardiovascular toxicity risk categories in cancer survivors based on the ESC Cardio-Oncology and ICOS Recommendations. Modified from [39,52]. DOX, doxorubicin; RT, radiation therapy; MHD, mean heart dose; Gy, gray.

ESC	
Childhood and adolescent cancer survivors	
Very high	RT > 25 Gy MHD (or prescribed RT ≥ 35 Gy to a volume exposing the heart) DOX ≥ 400 mg/m^2^ RT > 15 Gy MHD (or prescribed RT ≥ 35 Gy to a volume exposing the heart) + DOX ≥ 100 mg/m^2^
High	RT 15–25 Gy MHD (or prescribed RT ≥ 35 Gy to a volume exposing the heart) DOX 250–399 mg/m^2^ RT 5–15 Gy MHD (or prescribed RT 15–34 Gy to a volume exposing the heart) + DOX ≥ 100 mg/m^2^
Moderate	RT 5–15 Gy MHD (or prescribed RT 15–34 Gy to a volume exposing the heart) DOX 100–249 mg/m^2^ RT < 5 Gy MHD (or prescribed RT < 15 Gy to a volume exposing the heart) + DOX ≥ 100 mg/m^2^
Low	RT < 5 Gy MHD (or prescribed RT < 15 Gy to a volume exposing the heart) DOX 100–249 mg/m^2^
Adult cancer survivors	
Very high	Very high baseline CV toxicity risk pre-treatment Doxorubicin ≥ 400 mg/m^2^ RT > 25 Gy MHD (or prescribed RT ≥ 35 Gy to a volume exposing the heart) RT > 15–25 Gy MHD (or prescribed RT ≥ 35 Gy to a volume exposing the heart) + DOX ≥ 100 mg/m^2^
Early high	High baseline CV toxicity risk Symptomatic or asymptomatic moderate-to-severe CTRCD during treatment DOX 250–399 mg/m^2^ High-risk HSCT
Late high	RT > 15–25 Gy MHD (or prescribed RT ≥ 35 Gy to a volume exposing the heart) RT 5–15 Gy MHD (or prescribed RT 15–34 Gy to a volume exposing the heart) + DOX ≥ 100 mg/m^2^ Poorly controlled CVRF
Moderate	Moderate baseline CV toxicity risk DOX 100–249 mg/m^2^ RT 5–15 Gy MHD (or prescribed RT 15–34 Gy to a volume exposing the heart) RT < 5 Gy MHD or prescribed RT < 15 Gy to a volume exposing the heart) + DOX ≥ 100 mg/m^2^
Low	Low baseline CV toxicity risk and normal end-of-therapy cardiac assessment Mild CTRCD during therapy but recovered by the end of cancer therapy RT < 5 Gy MHD DOX < 100 mg/m^2^
ICOS	
High	Mediastinal radiotherapy ≥ 30 Gy with the heart in the treatment field Lower dose radiotherapy (<30 Gy) with anthracycline exposure Patients aged <50 years and longer time since RT High dose of radiation fractions (>2 Gy/d) Presence and extent of tumor in or next to the heart Presence of CV risk factors Pre-existing CV disease

## Data Availability

Data are available on request to corresponding author.

## Abbreviations

The following abbreviations are used in this manuscript:

3D-CRT	Three-dimensional conformal radiotherapy
AMC	Aorto-mitral curtain
AS	Aortic stenosis
CABG	Coronary artery bypass grafting
CCS	Childhood cancer survivor
CMR	Cardiac magnetic resonance
CS	Cancer survivor
CT	Computer tomography
CV	Cardiovascular
CVRFs	Cardiovascular risk factors
DIBH	Deep inspiration breath-hold
ECG	Electrocardiogram
ESC	European Society of Cardiology
EuroSCOREII	European System for Cardiac Operative Risk Evaluation II
Gy	Gray
HFA-ICOS	Heart Failure Association–International Cardio-Oncology Society
HL	Hodgkin lymphoma
ICOS	International Cardio-Oncology Society
IMRT	Intensity-modulated radiation therapy
LVOT	Left ventricular outflow tract
MAC	Mitral annular calcification
MHD	Mean heart dose
MR	Mitral regurgitation
MRT	Mediastinal radiotherapy
NPs	Natriuretic peptides
RT	Radiotherapy
SAVR	Surgical aortic valve replacement
STS-PROM	Society of Thoracic Surgeons Predicted Risk of Mortality
TAVI	Transcatheter aortic valve implantation
TEE	Transesophageal echocardiography
TEER	Transcatheter edge-to-edge repair
TMVR	Transcatheter mitral valve replacement
TTE	Transthoracic echocardiography
VHD	Valvular heart disease
VMAT	Volumetric modulated arc therapy
